# A Cost-Benefit and Accurate Method for Assessing Microalbuminuria: Single versus Frequent Urine Analysis

**DOI:** 10.1155/2013/752903

**Published:** 2013-12-25

**Authors:** Roholla Hemmati, Mojgan Gharipour, Alireza Khosravi, Mahnaz Jozan

**Affiliations:** ^1^Faculty of Medicine, Elam University of Medical Sciences, Elam, Iran; ^2^Isfahan Cardiovascular Research Center, Isfahan Cardiovascular Research Institute, Isfahan University of Medical Sciences, P.O. Box 81465-1148, Isfahan, Iran; ^3^Hypertension Research Centre, Isfahan, Iran

## Abstract

*Background*. The purpose of this study was to answer the question whether a single testing for microalbuminuria results in a reliable conclusion leading costs saving. *Methods*. This current cross-sectional study included a total of 126 consecutive persons. Microalbuminuria was assessed by collection of two fasting random urine specimens on arrival to the clinic as well as one week later in the morning. *Results*. In overall, 17 out of 126 participants suffered from microalbuminuria that, among them, 12 subjects were also diagnosed as microalbuminuria once assessing this factor with a sensitivity of 70.6%, a specificity of 100%, a PPV of 100%, a NPV of 95.6%, and an accuracy of 96.0%. The measured sensitivity, specificity, PVV, NPV, and accuracy in hypertensive patients were 73.3%, 100%, 100%, 94.8%, and 95.5%, respectively. Also, these rates in nonhypertensive groups were 50.0%, 100%, 100%, 97.3%, and 97.4%, respectively. According to the ROC curve analysis, a single measurement of UACR had a high value for discriminating defected from normal renal function state (*c* = 0.989). Urinary albumin concentration in a single measurement had also high discriminative value for diagnosis of damaged kidney (*c* = 0.995). *Conclusion*. The single testing of both UACR and urine albumin level rather frequent testing leads to high diagnostic sensitivity, specificity, and accuracy as well as high predictive values in total population and also in hypertensive subgroups.

## 1. Introduction

Chronic kidney disease imposes a large financial burden on patients and society. Early diagnosis and treatment of these disorders can slow progression to end-stage renal disease leading to lowering mortality and morbidity as well as reducing the overall costs [1]. Therefore, selection of cost-effective methods of diagnosing and screening for early chronic kidney diseases is needed to identify and manage subjects with this risky condition [2]. In this regard, following the presence of microalbuminuria as a sensitive marker especially in high-risk groups such as diabetics or hypertensive patients not only can provide valuable information on the stage of renal insufficiency but also can be cost-beneficial and prevent other diagnostic and therapeutic costs [3]. Assessment and screening for microalbuminuria is a confirmed useful tool for identifying subjects at risk for renal insufficiency and its progression. According to the this fact that the presence of microalbuminuria can be an alerting finding of increased risk for nephropathy and even cardiovascular disorders, a proper method for diagnosing and screening microalbuminuria should be considered to minimize the risk for these life-threatening events and related mortality and morbidity [4–6]. However, it is now controversy with regard to the type of urine test with acceptable cost-effectiveness and cost-beneficial to be used for evaluating microalbuminuria. The development of gold standard tests such as radioimmunoassay, immune-electrophoresis, and immune-turbidimetry method has provided the possibility for the measurement of even low concentrations of albumin in urine and its urinary secretion before the appearance of clinical warning manifestations [7]. However, because of its high cost and also inaccessibility to all segments of society particularly to residents in rural states, using this procedure repeatedly and also at different time points for accurate and definitive diagnosis is not applicable [8, 9]. In this regard, benefits and diagnostic accuracy of scheduling a single assessment of microalbuminuria and its cost-effectiveness in our country as a developing society remained unclear. Hence, we aimed to assess the cost-benefit of a single assessment of microalbuminuria especially in different subgroups at risk for developing renal failure.

## 2. Methods

The current cross-sectional survey was a part of a large comprehensive population-based cohort named “the Isfahan Healthy Heart Program” (IHHP) with the aim to identify subjects at risk for cardiovascular diseases, to control of cardiovascular risk profile, and to modify lifestyle [10]. In this study, a total of 126 consecutive persons were enrolled and interviewed at baseline for assessment of demographic parameters as well as hypertension state that was categorized hypertensive state if resting systolic blood pressure was ≥140 mm Hg and/or diastolic ≥90 mm Hg, or the subject was treated with antihypertensive medications. Because it was a part of a great population-based screening study named IHHP, we considered the consecutive persons who were scheduled for concurrent assessment of hypertension state and urine analysis. Thus, only those without complete data on hypertension status, those who did not agree with participation in the study (agreeing with blood pressure measuring or urinary examination) were not included into the study. The different stages of the test were performed by the trained and experienced personnel who were blinded to the purposes of the study. In this regard, only main researcher and our statistician informed the aims. The blood pressure was measured four times using a mercury sphygmomanometer, with an appropriate size cuff, and the mean of these measurements was considered as criteria for defining hypertension. During the measurements, the participant remained seated for 10 minutes with the arm comfortably placed at the level of the heart. In this study, those with the history or any evidences of diabetes mellitus, acute inflammatory disorders, trauma, major surgery, febrile disorders, connective tissue disorder, cerebrovascular accident, recent myocardial infarction, cardiac arrhythmias, chronic pulmonary diseases, malignancies, or heavy physical activities a day before the study begins were all excluded. The study protocol, which complies with the principles of good clinical practice and the declaration of Helsinki, has been approved by the relevant ethics committee at the participating center. Written informed consent was obtained from all participants before enrolment in the survey. Albuminuria was assessed by collection of two fasting random urine specimens on arrival to the clinic as well as one week later in the morning. Urine creatinine was measured by the picric acid method, and urine albumin content was measured by a sensitive, nephelometric technique (Pars Azmoon kits, Iran). The urinary albumin/creatinine ratio (UACR) and all other laboratory values were determined in a central laboratory within 24 hours after obtaining the urine samples. Microalbuminuria was defined as UACR from 30 to 300 mg/g in two consecutive portions taken a least 1 week apart [11]. According to the definition of microalbuminuria as albumin exceeding 30 mg/g in two consecutive samples, the benefits of one measurement of this parameter were assessed compared to the diagnosis on two first consecutive measurements as the gold standard. Kidney damage was also defined as UACR > or = 200 mg/g [12]. Results were reported as mean ± standard deviation (SD) for quantitative variables and percentages for categorical variables. We evaluated specificity, sensitivity, and positive (PPV) and negative (NPV) predictive values of measurement of microalbuminuria in a first-morning urine sample in comparison with the measurements in two timed urine collections as the gold. A receiver operating characteristic (ROC) curve was used to identify values of both two methods for measurement of microalbuminuria to discriminate normal from damaged kidney conditions. For the statistical analysis, the statistical software SPSS version 19.0 for windows (SPSS Inc., Chicago, IL) was used. *P* values of 0.05 or less were considered statistically significant.

## 3. Results

**Figure 1:**
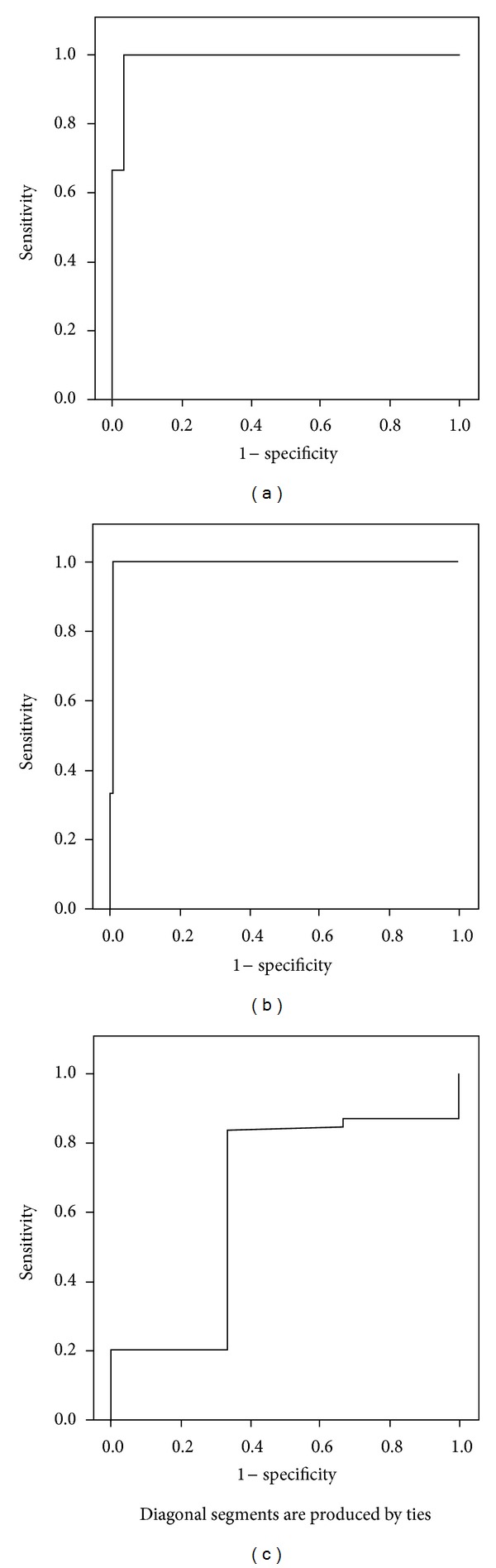
Receiver operator characteristic (ROC) curves to investigate the diagnostic power of a once UACR, urine albumin level, and urine creatinine level for predicting damaged kidney.

In overall and based on the diagnosis of microalbuminuria as UACR from 30 to 300 mg/g in two consecutive portions, 17 out of 126 participants suffered from microalbuminuria that, among them, 12 subjects were also diagnosed as microalbuminuria once assessing this factor with a sensitivity of 70.6%, a specificity of 100%, a PPV of 100%, a NPV of 95.6%, and an accuracy of 96.0% (Table 1). The measured sensitivity, specificity, PVV, NPV, and accuracy in hypertensive patients (*n* = 88) were 73.3%, 100%, 100%, 94.8%, and 95.5%, respectively. Also, these rates in nonhypertensive groups were 50.0%, 100%, 100%, 97.3%, and 97.4%, respectively. According to the ROC curve analysis (Figure 1), once measurement of UACR had a high value for discriminating defected from normal renal function state (*c* = 0.989, 95% CI: 0.967–1.000). Urinary albumin concentration in a once measurement had also high discriminative value for diagnosis of damaged kidney (*c* = 0.995, 95% CI: 0.982–1.000), while measurement of urine creatinine level had no acceptable value for discriminating damaged kidney function from normal function state (*c* = 0.638, 95% CI: 0.287–0.989).

## 4. Discussion

Several studies have demonstrated cost-effectiveness of microalbuminuria screening in high-risk populations leading optimization of patients' care as well as future implementation of CKD screening programs [8, 9, 13]. However, the limitations due to availability of instruments and also high costs for repeated measurement of biochemical markers in rural areas in most developing countries do not permit to the test in successive time periods. Inevitably, in these areas, one testing is preferred. However, the reliability of the test results may be questionable. The purpose of this study was to answer the question whether a single testing for microalbuminuria results in a reliable conclusion leading to costs saving. According to our observation, the once testing rather frequent testing leads to high diagnostic sensitivity, specificity, and accuracy as well as high predictive values in total population and also in hypertensive subgroups. On the other hand, the use of tests at different consecutive time points can be confidently replaced by the once testing with the partially same diagnostic value. Under the existing tariffs on testing microalbuminuria, the cost per test is estimated to be the equivalent of ten thousands of Rials that can be reduced by half using one testing. This cost saving can be quite substantial the entire community. Our purpose was not directly estimating and calculating the cost of urine analysis and comparing this cost between the single and repeated analyses. In fact, we attempted to point that because of high obtained accuracy by conducting single urine albumin analysis, it is not needed to repeat analyses and in this regard, this approach can certainly result in lower spending.

In our study, the diagnostic accuracy of the first-morning test for microalbuminuria was cost-benefcial for hypertensive patients as well as the total population. In fact, this once test can be beneficially used to screen microalbuminuria in both hypertensive and normotensive patients with the minimum cost. It has been previously shown that screening recommendations can be extended to include persons with hypertensive or diabetic patients [8, 13]. This is important because the bulk of the costs in these risk subgroups are spent for treatment and prevention of related complications. Thus, the use of one testing can be even more cost-benefcial in hypertensive patients with the same diagnostic accuracy.

Besides assessing the diagnostic accuracy of the one testing UACR for detection of microalbuminuria, we test diagnostic performance of one estimating urine albumin level and also urine creatinine level for assessment of kidney damage. According to the obtained discriminative values, the value of UACR and urine albumin level determination was equal, but measurement of urine creatinine level alone was notably low for this aim. Urine albumin is the key marker for chronic kidney disease. However, it is useful to compare the amount of albumin in the sample against its concentration of creatinine. The UACR has been shown to be convenient, cost-effective, and efficient in screening patients for microalbuminuria when compared with 24-hour collections [14, 15]. It seems that because UACR assessed the condition of urine albumin excretion based on the ability of kidneys to clear creatinine, the use of UACR marker is preferred to estimate urine albumin level alone for assessing severity of kidney damage.

The main limitation of the study was considering a partially small sample size leading to difficulties in high-power estimation of diagnostic accuracy (leading to a wide confidence interval especially for normotensive group) as well as diagnostic power measured by the ROC analysis. In another portion, considering other baseline variables especially socioeconomic level can help to explain more the affordability of the single testing. In addition, by considering different levels and severity of hypertension, assessment of affordability according to hypertension state can be achieved with more precision.

In conclusion, our results showed that the detection of both urinary albumin concentration and also estimation of UACR in single urine sample is accurate and cost-benefit procedure to identify microalbuminuric subjects regardless of hypertension state.

## Figures and Tables

**Table 1:** Details of accuracy analysis in total, hypertensive, and normotensive populations.

Population	TP	FP	FN	TN	Sensitivity	Specificity	PPV	NPV
Total	12	0	5	109	70.6% (44.0%–88.6%)	100% (95.8%–100%)	100% (69.9%–100%)	95.6% (89.6%–98.4%)
Hypertensive	11	0	4	73	73.3% (44.8%–91.1%)	100% (93.8%–100%)	100% (67.9%–100%)	94.8% (86.5%–98.3%)
Normotensive	1	0	1	36	50.0% (2.66%–97.3%)	100% (88.0%–100%)	100% (5.46%–100%)	97.3% (84.2%–99.9%)
